# Quantitative Modeling of Coupled Piezo-Elastodynamic Behavior of Piezoelectric Actuators Bonded to an Elastic Medium for Structural Health Monitoring: A Review

**DOI:** 10.3390/s100403681

**Published:** 2010-04-13

**Authors:** Guoliang Huang, Fei Song, Xiaodong Wang

**Affiliations:** 1 Department of Systems Engineering, University of Arkansas at Little Rock, Little Rock, AR 72204, USA; 2 Department of Applied Science, University of Arkansas at Little Rock, Little Rock, AR 72204, USA; E-Mail: fxsong@ualr.edu; 3 Department of Mechanical Engineering, University of Alberta, Edmonton, Alberta AB T6G 2G8, Canada; E-Mail: xiaodong.wang@ualberta.ca

**Keywords:** piezoelectric actuators, coupled piezo-elastodynamic behavior, elastic waves, structural health monitoring

## Abstract

Elastic waves, especially guided waves, generated by a piezoelectric actuator/sensor network, have shown great potential for on-line health monitoring of advanced aerospace, nuclear, and automotive structures in recent decades. Piezoelectric materials can function as both actuators and sensors in these applications due to wide bandwidth, quick response and low costs. One of the most fundamental issues surrounding the effective use of piezoelectric actuators is the quantitative evaluation of the resulting elastic wave propagation by considering the coupled piezo-elastodynamic behavior between the actuator and the host medium. Accurate characterization of the local interfacial stress distribution between the actuator and the host medium is the key issue for the problem. This paper presents a review of the development of analytical, numerical and hybrid approaches for modeling of the coupled piezo-elastodynamic behavior. The resulting elastic wave propagation for structural health monitoring is also summarized.

## 1. Introduction

Elastic waves, particularly guided waves (GWs), have shown great promise to identify damage in aerospace, aircraft and marine structures. In these applications, piezoelectric materials can be employed as actuators to generate high-frequency elastic waves that carry the structural information, based on the converse piezoelectric effect [1–4]. Building a model for the structures integrated with piezoelectric actuators (piezo-actuators) to understand their electromechanical dynamic behavior and simulate their resulting wave propagation is a prerequisite for the design and optimization of elastic wave based-structural health monitoring (SHM) systems. Piezoelectric materials attached to or embedded in structures may largely influence local structural behavior. The efficiency of actuation is related to not only the material properties of piezoelectric materials but also those of the host structure and the applied loading frequency. The most important parameters should be identified and analyzed to qualify the proposed actuators technology [5–7]. Due to the presence of the material discontinuity between the actuators and the host structure, a complicated stress field is generated, especially for the position near the edges of the actuators, where stress concentration occurs. For example, the induced stress concentration near the tips of an actuator may result in undesired peeling-off of the actuator from the host structure, which may result in a reduction of the load transfer capability of the structure, and hence the actuator may lose its ability to perform its role [8–10]. An accurate assessment of the coupled electromechanical behavior of piezoelectric structures would, therefore, necessitate the detailed study of the load transfer between the piezo-actuators and the host structure.

To avoid the difficulties associated with the complicated interfaces between the actuators and the host medium, some simplified actuator models have been used to simulate the actuation process of embedded and bonded thin sheet actuators. The uniform strain model was first developed for a cantilever beam with a layer of PVDF bonded on one side only [11]. The modeling was based on force equilibrium between the actuator and the beam. A constant actuator force output proportional to the applied voltage was obtained. A more extensive model was later proposed by Crawley and his coworkers [12,13] to analyze a beam-like structure with bonded and embedded thin sheet piezoelectric actuators to study the load transfer between the actuators and the host beam. In this analysis, the axial stress in the actuator was assumed to be uniform across its thickness and the host structure was treated as a Bernoulli-Euler beam. Im and Atluri [14] modified the actuator model presented by Crawley and de Luis [12] by considering both the axial and the transverse shear forces in the beam. A refined actuator model based on a second order axial normal stress field was presented for a beam structure with symmetrically bonded actuator patches [15,16]. This model was based on the plane stress formulation and solved by the principle of stationary complementary energy. Richard and Cudney [17] presented an analytical model for multiple layer piezoelectric actuators in which Timoshenko's beam theory led to equations of motion for lateral vibration that included rotary inertia and shear deformation effects. Twist, shear and torsion can be generated for the piezoelectric actuator applied to an anisotropic composite structure. An integrated theory was used to model the bending/twisting/shearing actuation of laminated beams [18].

Plate and shell models have also been extensively developed in modeling the piezoelectric structures. Lee and Moon [19] applied the classical laminate plate theory to the design of piezoelectric laminate for bending and torsion modal control. An analytical model for multi-layered thin shells with distributed piezoelectric actuators was proposed by Tzou and Gadre [20]. In the work, the theoretical development was based on Love’s thin shell theory in which the transverse shear deformation and the rotary inertia were neglected, and the governing equations were established based on Hamilton’s principle. A consistent plate model was developed by Crawley and Lazarus [21]. This model is a simple extension from the one-dimensional beam model to the two-dimensional plate model. Wang and Rogers [22] modified the classical laminated plate theory to model actuator-induced bending and extension of laminated plates under static loading. This work provided a theoretical basis of general application of induced strain actuators. The vibration control of a simply supported rectangular plate was studied by Batra *et al.* [23]. Thin layers of PZT ceramic were attached to the top and bottom of the rectangular plate, which was assumed to be vibrated at a frequency close to one of its natural frequencies, to achieve the control. For structures with curvatures such as rings and shells, analytical models based on layered shell theory have been proposed to consider the coupling between the in-plane and out-of-plane displacements. An analytical model for thick composite piezoelectric shells was proposed by Tzou and Zhong [24]. Other typical examples for modelling piezoelectric actuators include the works in [25–40]. For more complex structures, analytical modelling becomes strenuous, and numerical methods, such as finite element analysis, should be considered to resolve such problems.

The beam and plate models operate with the lowest fundamental (bending and longitudinal) guided wave modes, and thus they provide a simple description of the elastodynamics wave processes in the host structure. Their application is, however, restricted, since they are valid only in a low frequency range where the characteristic wavelength is much greater than the plate or shell thickness. Therefore, models based on the Rayleigh-Lamb equations for an elastic layer and/or a half-space have been attracting much attention to capture the high-order Lamb wave modes or Rayleigh surface waves. Giurgiutiu [41] proposed a simplified analytical model for an isotropic plate under plane strain assumptions to obtain the harmonic Lamb wave responses. In the study, the interfacial shear transfer was assumed to be localized only at the tips of the actuator as the simplified pin-force model. Following the similar theoretical procedure, Raghavan and Cesnik [42] has extended the two-dimensional model to the three-dimensional elasticity model for bonded circular and rectangular piezoelectric actuators. Double Fourier transform was used to obtain high-frequency Lamb wave propagation. In the work, both harmonic and transient wave responses were studied and the predicted waveforms were found to agree well with the experimental results in shape for low frequency cases. In above studies, the actuation mechanism of the actuators is still based on the assumption that actuation shear traction exerted by the actuators is independent of frequency. The coupled piezo-elastodynamic behavior between the actuator and the host medium is not considered to provide the quantitative dynamic interfacial stress distribution and its resulting wave propagation.

Various numerical simulation tools, such as commercially available finite element (FE) codes, have allowed users to conduct coupled multi-physical field solutions in a relatively convenient way. Nieuwenhuis *et al.* [43] simulated guided wave generation, propagation and reception in an isotropic plate bonded with PZT (lead-zirconate-titanate) wafers by the FE modeling. The coupled electrostatic solution was analyzed. However, the FE simulation has its own limitations for large structures, since it operates within spatially restricted discretization. Hybrid numerical-analytical approaches provide an alternative solution for this problem, where numerical simulation is only performed to treat the coupled piezoelectricity problem. The interfacial stresses due to the piezoelectric element are numerically obtained as prescribed excitation, and the resulting wave propagation in the host structure is then analytically described. Following this idea, Moulin *et al.* [44–46] proposed a hybrid approach to model integrated Lamb wave generation with piezo-acuators/sensors. A coupled finite element-normal modes expansion method was used for the simulation of piezoelectrically induced Lamb wave propagation.

Compared with the numerical simulation, the analytical approach to consider the coupled dynamic behavior can give quantitative study of the interfacial stress and its resulting wave propagation. Liu *et al.* [8] studied the static shear stress distribution between a partially electroded thin piezoelectric film and a semi-infinite elastic substrate. In the study, the shear stress was governed by a pair of integro-differential equations. An integral equation based model for a system of piezoelectric flexible patch actuators bonded to an elastic substrate (layer or half-space) was proposed to consider the interaction between piezoelectric patches and the host medium [47]. An analytical formulation to couple actuators dynamics with axisymmetric guided wave excitation models for an isotropic plate was recently reported [48]. In the work, the piezo-actuator was modeled using coupled piezoelectricity-elasticity equations. The actuation mechanism was represented by the interfacial shear force only at the tips of the actuator. The amplitude of the shear force was calculated by matching the traction and displacement at the actuator’s edge with the same position of the structure. All possible guided wave modes were considered and the limitation of the model for high-frequency guided waves was also discussed. Wang and Meguid [49] developed a one-dimensional actuator model to examine the static coupled electromechanical behavior of a thin piezoceramic actuator embedded in or bonded to an elastic medium under in-plane mechanical and electrical loadings, in which the load transfer and the local stress field around the actuator were studied. This actuator model was further extended and modified by Wang and Huang [50] to consider dynamic electromechanical behavior of actuators bonded to and/or embedded in elastic half-space. The advantage of the proposed model is that the local interfacial stress distribution between the actuator and the host medium can be fully captured, even for high wave frequencies. The harmonic wave propagation generated by bonded and embedded piezoelectric actuators was then analytically studied and the interaction between multiple actuators was also simulated by using the developed pseudo-incident wave method [50].

This paper presents a comprehensive review on the state of the art of modeling techniques for piezoelectric wafer actuators bonded to the elastic medium, particularly some representative analytical, numerical and hybrid approaches to model the coupled piezo-elastodynamic behavior, and some resulting ultrasonic wave phenomenon and applications relevant to SHM are also summarized.

## 2. Review of the Bonded Piezo-Actuator Models

In this section, the approaches that aim to achieve the coupled electromechanical behavior of the piezo-actuators bonded to the host structure are reviewed and summarized. These methods include both analytical, numerical and hybrid schemes.

### 2.1. Analytical Approaches

#### 2.1.1. The shear-lag theory based on the Euler-Bernoulli model

**Figure 1.**
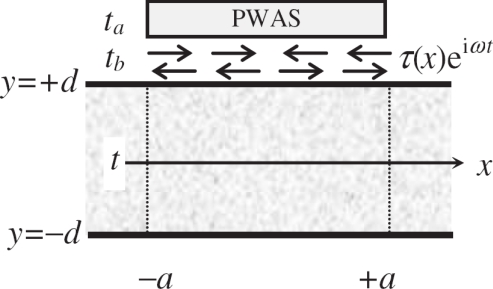
Illustration of a piezoelectric wafer actuator bonded to the host structure. Taken from Giurgiutiu [41].

The Euler-Bernoulli model is one of the earliest models developed for beams actuated by the piezoelectric wafers. The widely used analytical model [12] was first developed to obtain the interfacial shear stress by using shear-lag theory. In their work, the configuration of two thin piezoelectric elements bonded on both sides of the elastic beam was studied. In the model, only non-zero stress within the adhesive layer is the interfacial shear stress, which is assumed as constant through the thickness of the adhesive layer and varies along the longitudinal direction of the adhesive layer. The actuator is modeled as a beam with only the axial stiffness, whereas the passive beam is modeled as an Euler-Bernoulli beam. Basically, the model assumes (i) uniform strain in the bonded piezo-actuator, and (ii) uniform strain for axial motion and linear strain distribution for flexural motion across the thickness in the beam. A shear-lag solution can be then derived for the static interfacial stress between the piezoelectric actuator and the beam as:

(1) $$\tau (x)=\frac{t_{a}}{a}\frac{\psi }{\alpha +\psi }E_{a}\varepsilon_{a}\left(\Gamma a\frac{\text{sinh}\;\Gamma x}{\text{cosh}\;\Gamma a}\right)$$

where the stiffness ratio between the piezoelectric actuator and the host structure is $\psi =\frac{Et}{E_{a}t_{a}}$, and *ε_a_* denotes the induced strain by the piezo-actuator. The shear-lag parameter $\Gamma^{2}=\frac{G_{b}}{E_{a}}\frac{1}{t_{a}t_{b}}\frac{\alpha +\psi }{\psi }$ indicates the effectiveness of the shear transfer. *Γ* is affected by the stiffness and thickness of the bonding layer *t_b_*. This initial work was further extended by Crawley and Aderson [13] to illustrate the extension, bending, and localized shear deformations induced. The shear-lag parameter *Γ* also depends on a constant *α*, and *α* relies on the stress and strain distribution across the beam thickness. If two piezoelectric actuators are installed, and only extensional wave motion is generated, then *α* = 1; if only the flexural wave motion is generated, then *α* = 3. By calculating the total effect as a superposition of symmetric and anti-symmetric contributions, *α* = 4 was found for a single-sided piezoelectric wafer actuator configuration [41] as shown in Figure 1, where both extensional and flexural wave motion are equally excited. Crawley and de Luis’s model [12] can be also enhanced by assuming linear strain distribution in both the actuator and the beam, and thus the flexural stiffness of the actuator can be considered in the resulting global behavior of the system [51], which may become more important for modeling thicker actuators. Some other modifications include the extension of Euler-Bernoulli beam models to Timoshenko beam models considering the shear deformation and rotary inertia, and the extension of one-dimensional beam models to two-dimensional plate models [52–54].

However, the shear-lag theory has its intrinsic limitations: (i) the theory assumes linear strain distribution across the beam thickness, and this approximation only applies for low values of the frequency-thickness product of the lowest symmetric (S_0_) and anti-symmetric (A_0_) modes, and (ii) the theory cannot capture more than two lowest S_0_ and A_0_ modes with the increase of frequency.

To overcome these critical limitations, Giurgiutiu and Bottai-Santoni [55] extended the classic shear-lag theory [12] by taking into account the nonlinear stress distribution along the beam thickness for the S_0_ and A_0_ modes as:

(2) $$\sigma (x,y)=a_{S}(x)\sigma_{S}(y)+a_{A}(x)\sigma_{A}(y)$$

Where *σ_s_* (*y*) and *σ_A_* (*y*) are the stresses induced by the nonlinear S_0_ and A_0_ modes, respectively, and *σ_s_* (*x*) and *σ_A_* (*x*) are *x*-dependent modal participation factors. Following the similar manner, the value of the parameter *α* can be then derived as:

(3) $$\alpha ={\left(\Lambda_{S}\right)}^{-1}\sigma_{S}(d)+{\left(\Lambda_{A}\right)}^{-1}\sigma_{A}(d)$$

where $\Lambda_{S}=\frac{1}{t}\int_{-d}^{+d}\sigma_{S}(y)\mathrm{dy}$, and $\Lambda_{A}=\frac{1}{\mathrm{td}}\int_{-d}^{+d}\sigma_{A}(y)\mathrm{ydy}$.

Equation (3) can be regarded as a direct extension of Crawley and de Luis’s work [12] in which the value of *α* is dependent on low-frequency beam theory assumption and is not necessarily applicable to high frequency times plate thickness product.

#### 2.1.2. The simplified pin-force model

**Figure 2.**
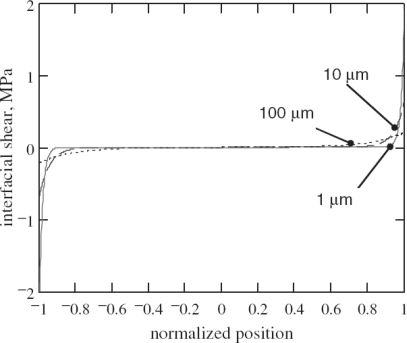
Variation of interfacial shear stress with respect to bond thickness. Taken from [41].

When the bonding layer becomes thinner and stiffer, Crawley and de Luis’s [12] and Giurgiutiu [41] demonstrated that the interfacial shear stress transferred from the piezo-actuator to the host structure could confine more at the edges of the actuator as shown in Figure 2.

For a perfectly bonded actuator with the limiting case of an infinitely stiff bonding layer, the shear lag parameter *Γ* approaches infinity. In this case, a sharp rise in the shear stress exists at the tips of piezo-actuator, indicating that the strain is transferred between the piezo-actuator and the host structure over an infinitesimal distance near the edge of the actuator. These idealized assumptions yield the concept of the simplified pin-force model [41]. Consequently, the shear stress distribution along the actuator-host structural interface was expressed using Dirac function *δ* (*x*) as [41]:

(4) $$\tau_{a}(x)=\tau_{0}[\delta (x-a)-\delta (x+a)]$$

where *τ_0_* is the pin force magnitude applied at the piezoelectric actuator edges.

The two-dimensional pin-force model for the bonded piezo-actuator was proposed in [42] as:

(5) $$\tau_{31}(x)=\tau_{0}[\delta (x_{1}-a_{1})-\delta (x_{1}+a_{1})][H_{e}(x_{2}+a_{2})-H_{e}(x_{2}-a_{2})]$$

(6) $$\tau_{32}(x)=\tau_{0}[H_{e}(x_{1}+a_{1})-H_{e}(x_{1}-a_{1})][\delta (x_{2}-a_{2})-\delta (x_{2}+a_{2})]$$

The simplified pin-force model can be readily applied in the host structure as traction boundary conditions to obtain the dynamic response of the system. Under plain strain assumption, Giurgiutiu [41] coupled the pin-force model with the Rayleigh-Lamb equations [56] to simulate the resulting harmonic guided wave propagation in an isotropic plate. Raghavan and Cesnik [42] extended Giurgiutiu’s work [41] by using the three-dimensional elasticity theory and conducted transient analysis for the induced guided wave signals. Lin and Yuan [57] studied diagnostic transient waves in an infinite isotropic plate generated by a pair of bonded circular actuators. The actuation mechanism was represented by bending moments along the actuator edges, which is similar as the concept of the simplified pin-force model.

Major limitations of the simplified pin-force model are summarized as follows: (i) The model is a good approximation only if the Young’s modulus and thickness of the actuator are small compared to those of the host structure or the bonding layer is very thin and stiff, (ii) the model can only provide qualitative estimation about the actuation mechanism for low-frequency cases, which needs to be calibrated by either numerical simulation or experimental testing, and (iii) piezoelectric resonance effects cannot be captured in the model [48].

**Figure 3.**
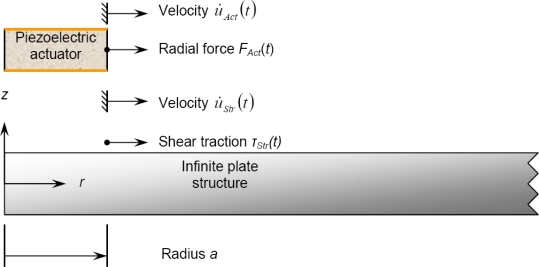
Free body diagram of the actuator/host structural system. Taken from Dunn *et al.* [48].

Recently, Dunn *et al.* [48] attempted to couple actuator dynamics with axisymmetric guided wave excitation model for isotropic plates. In the work, the piezo-actuator dynamics are modeled using piezoelectricity-elasticity equations, and bonded-actuator is assumed to cause shear traction on the structural substrate along the actuator edge. The free body diagram of this approach is shown in Figure 3, in which a thin piezoelectric disk is driven on the edge using a radial force *F_Act_* (*t*), with a resulting velocity *u̇_Act_*(*t*) and excited with a voltage *V_Act_* (*t*) with a induced current *i̇_Act_*(*t*).

The relationship between the edge force, edge velocity, voltage, and current for the piezo-actuator in the spectral domain can be expressed as [48]:

(7) $$\left\{\begin{array}{c} F_{\mathrm{Act}}(\omega )\\ V_{\mathrm{Act}}(\omega ) \end{array}\right\}=\left[\begin{array}{cc} Z_{11}^{\mathrm{Act}}(\omega ) & Z_{12}^{\mathrm{Act}}(\omega )\\ Z_{12}^{\mathrm{Act}}(\omega ) & Z_{22}^{\mathrm{Act}}(\omega ) \end{array}\right]\left\{\begin{array}{c} {\dot{u}}_{\mathrm{Act}}(\omega )\\ {\dot{i}}_{\mathrm{Act}}(\omega ) \end{array}\right\}$$

where the impedance $Z_{ij}^{\mathrm{Act}}\left(i,j=1,2\right)$ is related to the planar piezoelectric material properties. An outward radial force *F*_Act_ is assumed to apply on the edge of the actuator, which leads to a velocity at the actuator edge *u̇_Act_*. On the top surface of the structure at *r* = *a* is the shear traction *τ_Str_*, which results in the reaction velocity of *u̇_Str_*. Summation of forces at the actuator edge yields:

(8) $$F_{\mathrm{Act}}(t)=-2\pi a\;\tau_{\mathrm{Str}}(t)$$

The application of the continuity of the displacement at the edge leads to:

(9) $${\dot{u}}_{\mathrm{Act}}(t)={\dot{u}}_{\mathrm{Str}}(t)$$

However, this model is still based on the pin-force model, which cannot capture the electromechanical interfacial stress distribution between the actuator and the host structure, especially for high-frequency cases.

#### 2.1.3. The elasticity equation-based model

**Figure 4.**
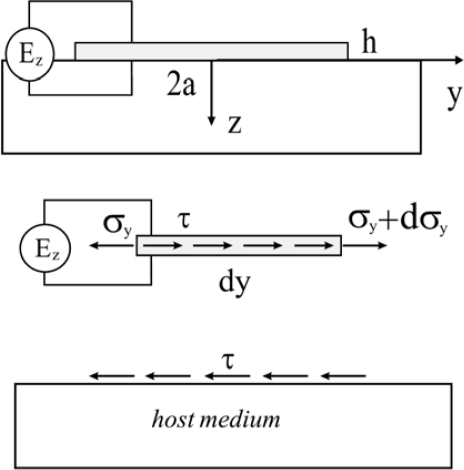
The actuator model with coupled piezo-elastodynamics. Taken from Huang and Sun [66].

One major disadvantage of using plate/beam theory is that it can only approximately model the lowest A_0_ Lamb wave modes when the excitation frequency-plate thickness produce is sufficiently low. Therefore, the models employing the Rayleigh-Lamb equations for the elastic host medium attracts more attention to consider high-frequency Lamb waves and Rayleigh surface waves [58–60]. Under the plain strain assumption, Lanza di Scalea and Salamone [61] coupled shear-lag theory of Crawley and de Luis [12] with the Rayleigh-Lamb equations as traction boundary condition and obtained the piezoelectrically induced Lamb waves in the plate. However, due to the limitation of the shear-lag solution [12], this model is also not suitable for the high frequency times plate thickness products. To consider the coupled piezo-elastodynamic behavior, Wang and Huang [50, 62–65], and Huang and Sun [66] developed an one-dimensional electroelastic actuator model bonded to the elastic half-space medium. In the model, the geometry, loading frequency and material combination effects on the interfacial shear stress were captured. Therefore, the model can provide the quantitative prediction of dynamic load transfer. The illustration of this model is plotted in Figure 4.

The solution of the host structure is based on the elasticity theory. In the model, the actuator thickness is assumed to be very small in comparison with its length, the applied electric filed primarily results in an axial deformation, and the following assumptions can be made: (i) *σ_y_* and *u_y_* are uniform across the thickness of the actuator; (ii) the interfacial shear stress (*τ*) transferred between the actuator and the host can be replaced by a distributed body force along the actuator, and (iii) *σ_z_* and *σ_vz_* in the actuator can be ignored. Also, bonding layer effects between the actuator and the host medium are neglected in this model. Based on these assumptions, the equations of motion of the actuator along the axial direction under plane strain analysis can be expressed as:

(10) $$\frac{d\sigma_{y}^{a}}{\mathrm{dy}}+\frac{\tau (y)}{h}+\rho_{a}\omega^{2}u_{y}^{a}=0$$

where the superscript “*a*” represents the actuator, *h* is the thickness of the actuator, *ρ_a_* is the mass density of the actuator, and *ω* is the circular loading frequency. The axial strain of the actuator can be then obtained in terms of the interfacial shear stress *τ* by solving Equation (10) as:

(11) $$\varepsilon_{y}^{a}=\varepsilon_{E}(y)+\frac{\text{sin}k_{a}(a+y)}{{hE}_{a}\text{sin}2k_{a}a}\int_{-a}^{a}\text{cos}k_{a}(\zeta -a)\tau (\zeta )d\zeta -\int_{-a}^{y}\text{cos}k_{a}(\zeta -y)\frac{\tau (\zeta )}{hE_{a}}d\zeta$$

where *E_a_* and *e_a_* are effective elastic and piezoelectric material constants [50], $\varepsilon_{E}\left(y\right)=\frac{e_{a}E_{z}}{E_{a}}\frac{\text{cos}k_{a}y}{\text{cos}k_{a}a}$, *k_a_* = *ω*/*c_a_*, and $c_{a}=\sqrt{E_{a}/\rho_{a}}$ with *k_a_* and *c_a_* being the wave number and the axial wave velocity of the actuator, respectively. The continuity between the actuator and the host structure at z = 0 can be described as:

(12) $$\varepsilon_{y}^{a}(y)=\varepsilon (y,0),|y|<a$$

where *ε* (*y*,0) is the induced surface elastic strain in the host medium in the actuation area. Equation (12), which is used to couple the actuator dynamics with the structural dynamics, results in a first kind of singular integral equation involving a square-root singularity of *τ* at the tips of the actuator. The general solution of *τ* can be solved using Chebyshev polynomial expansions. The induced wave propagation in the host medium can be obtained by using elasticity equations [50]. The advantage of this developed model is that it includes the coupled dynamic interaction between the actuator and the host medium, and hence can quantitatively predict the piezoelectrically induced electromechanical behavior.

**Figure 5.**
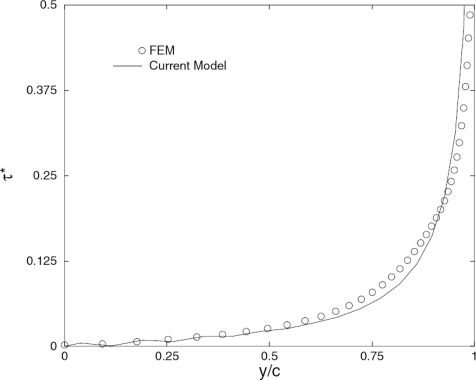
The normalized interfacial shear stress. Taken from Wang and Huang [50].

**Figure 6.**
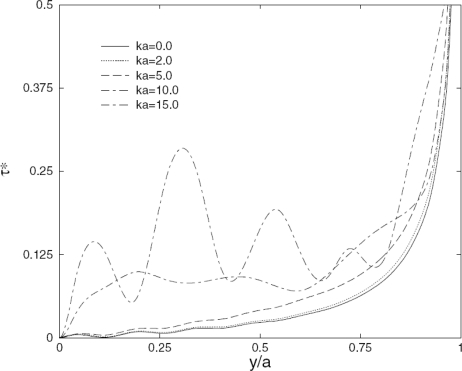
The loading frequency effects on the normalized interfacial shear stress. Taken from Wang and Huang [50].

**Figure 7.**
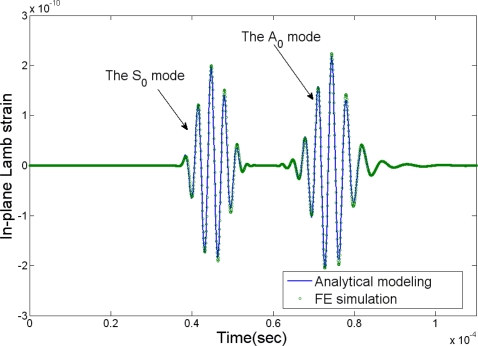
The comparison of the resulting Lamb waves predicted by the integral model [50] and the finite element simulation.

Figure 5 shows comparison of the normalized static interfacial shear stress distribution along the interface between the actuator and the infinite host medium predicted by the developed model with that obtained from FE analysis using ANSYS/Multiphysics [50]. Very good agreement between the current model and FE simulation is observed in the figure. Figure 6 demonstrates the load frequency influences on the normalized dynamic shear stress distribution predicted by the developed model (*ka* is the normalized wavenumber), and the significant effect of the loading frequency upon the interfacial shear stress can be observed. So it is very important to consider this coupling effect especially for the cases with high frequency times plate thickness products. Figure 7 shows the comparison of the resulting Lamb waves predicted by the integral model [50] and the finite element method. In the simulation, PZT4 actuator is bonded to the aluminum plate. The excitation is 300 kHz five-peak tone burst ultrasonic signal, and the response is calculated at distance 226.3 mm away from the actuator. As evidenced in the figure, both the S_0_ and A_0_ modes predicted by the integral model [50] have an excellent agreement with the FE simulation results in phase and magnitude, which verifies the capability of the current actuator model for the resulting guided wave simulation.

Similar approaches to consider the coupled electromechanical behavior can be also found in the literatures [8,47,67].

### 2.2. Numerical and Hybrid Approaches

**Figure 8.**
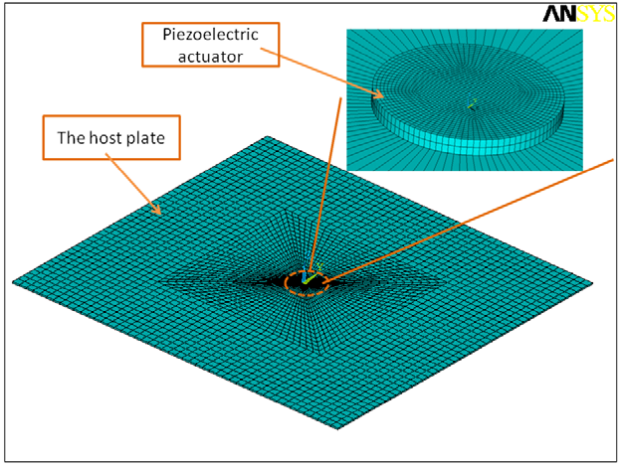
FE modeling and meshing of a circular piezo-actuator bonded to a host plate.

Numerical simulation techniques have been widely utilized to analyze the elastic wave behavior induced by the piezo-actuators [43,68,69]. In modeling the electromechanical interaction between the actuator and the host structure, some commercially available FE codes, e.g., COMSOL/Multiphysics and ANSYS/Multiphysics, provide researchers convenient tools to conduct the coupled physical problem. Figure 8 shows an example FE modeling and meshing of a circular piezo-actuator bonded to a plate structure using ANSYS/Multiphysics, in which the SOLID5 element with eight nodes and six degree of freedoms (DOF) at each node is selected for the piezo-actuator, and the SOLID45 element is used to model the plate structure. The additional DOF in this coupled field element is electrical voltage. Input voltage can be applied on the top nodes of the piezo-actuator, and zero voltage is usually assigned for all the bottom nodes of the piezo-actuator to simulate the grounding operation. The disadvantages of FE simulation are summarized as: (i) classical FE analysis cannot be directly applied to simulate infinite open waveguides, since it works within spatially restricted discretization domains [47], (ii) FE simulation lacks of the capability to provide a very clear physical explanation of the numerically predicted results, and (iii) coupled filed analysis may become extremely burdensome in computational effort for solving the responses of three-dimensional large structural models at high frequency, since a huge number of elements (at least ten elements per wavelength) are usually required to guarantee the numerical convergence.

**Figure 9.**
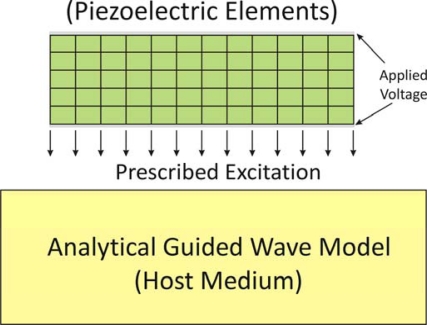
Illustration of the scheme of the hybrid approach.

Hybrid approaches provide potential solutions to compensate for the disadvantages of pure FE simulation. In the hybrid schemes, the FE solution using piezoelectric elements is only conducted in limited areas (e.g., the piezo-actuation area) to obtain the prescribed excitation, and then combined with analytical guided wave excitation model in the host structure, as shown in Figure 9. In the approach, the FE calculation is conducted to determine only the surface stresses or the volume forces created by the piezoelectric elements, which are used as the prescribed excitation for the analytical solution in the host medium [44–46]. The hybrid schemes enable the calculation of piezoelectrically induced wave response in the infinite host medium with less computational effort, since the host structural model usually consumes much more elements than does the piezo-actuator model. However, like FE simulation, this approach still lacks of the capability to provide a very clear physical explanation of the predicted results, especially connection between the prescribed excitation and its resulting wave propagation which is important for the SHM design and optimization.

## 3. Structural Health Monitoring Application

### 3.1. Dynamic Responses of the Host Structure Induced by the Piezo-Actuator

The most fundamental issue surrounding the effective use of piezo-actuators in SHM is the evaluation of the generated wave propagation. Based on the solution of the interfacial stress, the local dynamic response of the host medium generated by the piezo-actuator can be solved by using Fourier transform technique and solving the resulting integral equations in terms of the interfacial stress.

**Figure 10.**
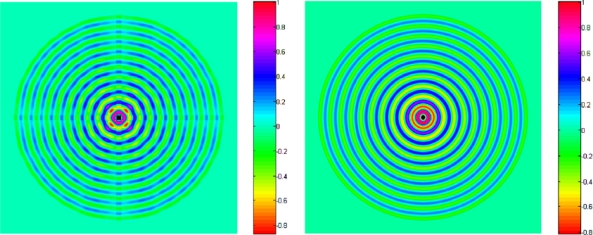
Downward view of normalized harmonic radiation field for out-of-plane surface displacement in an aluminum plate at 100 kHz, the A_0_ mode by a pair of (a) (left) square actuators and (b) (right) circular actuators. Taken from Raghavan and Cesnik [42].

**Figure 11.**
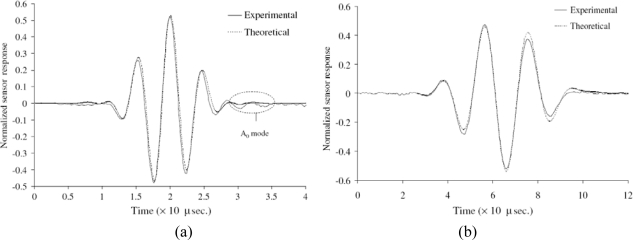
Comparison between the normalized sensor signals obtained analytically and experimentally for the circular actuator: (a) the S_0_ mode at central frequency of 200 kHz and (b) the A_0_ mode at central frequency of 50 kHz. Taken from Raghavan and Cesnik [42].

For its simplicity, the pin-force model [41,42] has been extensively used to predict the elastic wave fields induced by the piezo-actuator in isotropic or composite plates [41,42,60,70,71]. The representative work among them was conducted by Raghavan and Cesnik [42] based on the three-dimensional linear elasticity theory, and the piezo-actuator was modeled to induce uniform magnitude in-plane traction along its perimeter. Figure 10 shows the harmonic out-of-plane displacement patterns due to excitation of the A_0_ Lamb wave mode at 100 kHz in an aluminum plate by rectangular and circular actuators. It is found that the wave field excited by a rectangular actuator tends to a circular crested wave filed with angularly dependent amplitude at large distances from the actuator, while the circular actuator wave filed spatially attenuates with equally spaced peaks and troughs in the far field. The transient responses to a time-limited signal can be obtained by conducting the inverse Fourier transform of the integral of the product of the harmonic response [42]. Figure 11 displays the comparison of normalized theoretical and experimental sensor signals at certain central frequencies in the time domain [42].

### 3.2. Wave Mode Tuning with Piezo-Actuators

**Figure 12.**
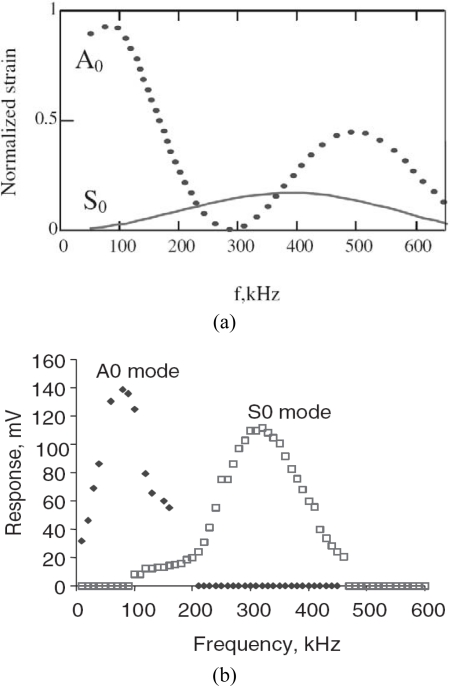
Lamb wave mode tuning with varying excitation frequencies (a) the simplified pin-force model and (b) the experimental testing. Taken from Giurgiutiu [41].

From the point of view of guided wave based SHM, the tuning of a particular mode is quite important, since it allows researchers to address the detection of specific damages with specific wave modes. To find the Lamb strain induced in the plate, Giurgiutiu [41] showed that it is possible to tune wave modes through the maxima and minima of the sin *ka* function with *ka* being the normalized wave number. Two important factors for the design of piezo-based guided wave SHM were further demonstrated: (i) the variation of |sin *ka*| with frequency for each Lamb wave mode, and (ii) the variation of the surface strain with frequency for each Lamb wave mode. Figure 12 shows the Lamb wave tuning realization in a 1.6 mm aluminum plate by using both the actuator model [41] and the experimental testing for the frequency range up to 600 kHz [41]. It can be found that theoretical prediction of the frequency tuning trend is fairly consistent with the experimental observation. It can be also seen that the A_0_ mode is excited very strongly at low frequencies, while the S_0_ mode is barely observed. A preferential excitation spot of the S_0_ mode can be identified around 300 kHz for the current actuator/structural configuration. More detailed description was given in refs. [41,60].

### 3.3. Quantitative Evaluation of Damage Using Elastic Waves

Elastic waves have been successfully used in the nondestructive evaluation (NDE) of materials and structures. Since elastic guided waves are sensitive to the material parameters of the host medium and can propagate over long distance, they can be used to detect surface/embedded damages in structures [72]. Many researchers have attempted to propose various methods to investigate the change in the data of sensors due to the damages and illustrate the possible detection of the presence of the damage [73–80]. Preliminary method of interpreting wave signals is to directly compare characteristic parameters of the signals from a structure with that of the virgin structure to identify potential damage. These parameters could be wave speed, arrival time, amplitude, attenuation, *etc.*, either in time domain or in frequency domain [81]. Giurgiutiu [82] used Lamb wave technique to compare amplitude changes in thin aluminum aircraft skins after various levels of usage to detect changes, and utilized finite element technique to attempt to predict the level of damage with some success. Su and his coworkers [79,83] utilized the time of flight (TOF) between the incipient fundamental symmetric Lame waves and delamination-induced fundamental shear horizontal mode to triangulate the delaminations in composite laminates. Using modally selective Lamb wave transducers, Petculescu *et al.* [77] demonstrated that the accumulated time delay of modal group velocity may be a reliable damage parameter for quantitative monitoring of delaminations for quasi-isotropic woven and cross-ply composites.

To establish the quantitative relation between the surface signals and the embedded damages in materials, efforts have been made both theoretically and experimentally by “propagating” elastic waves back to the damages from the surface. The idea is based on that the wave field is reversible [84,85]. Thus if one is able to use sensors to record a complete scattering wave field and find a method to back propagate the recorded waves, the energy of these waves will progressively converge back to the scattering source and indicates the existence of damage. Migration is a geophysics exploration technique to form the image of subsurface reflectors by moving or “migrating” the recorded wave field to their actual spatial locations, topology of the earth’s interior. Over the past thirty years, research on the migration technique has attained a maturity and is indispensable as an advanced interpretation method for reflection wave field [86,87]. Lin and Yuan [88,89], and Wang and Yuan [90] performed prestack reverse-time migration technique to image the damage in isotropic plate/composite laminates with a linear PZT disk array, and both the location and size of the damage were quantitatively obtained.

**Figure 13.**
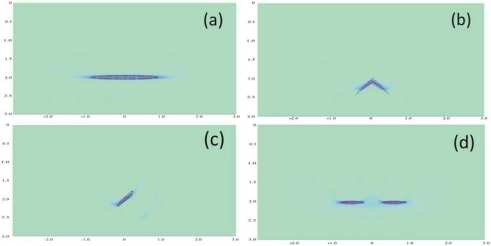
Image of embedded cracks with various shapes. (a) The linear crack (b) the wedge crack (c) the inclined crack and (d) two collinear cracks. Taken from Wang and Huang [91].

Similarly, a reverse wave technique using high-frequency piezo-induced elastic bulky wave propagation was presented to interpret the received elastic wave signals and detect embedded cracks [91,92] in the elastic medium. In the study, FE was used to simulate the elastic wave propagation in the cracked elastic medium with tone burst excitation applied on the piezo-actuator. As shown in Figure 13, the final image of the structure can predict not only the position of the crack with complicated shape but also the dimension of it. Moreover, it is demonstrated that multiple embedded cracks can be also visualized with a high resolution, which facilitates the application of this technique to interpret the elastic wave signals collected in the practical SHM systems.

## 4. Conclusions and Summary

Among the various schemes being considered for SHM, elastic waves generated by piezoelectric actuators have particularly shown great promise. In these applications, piezoelectric materials are usually employed as actuators to generate the high-frequency diagnostic elastic waves. To effectively use bonded piezo-actuators in these integrated SHM system, the quantitative evaluation of the induced elastic wave propagation is strongly needed. Accurate characterization of the coupled piezo-elastodynamic behavior between the actuator and the host medium is the key issue for the problem. This paper reviews the state of the art and recent advance of different modeling approaches for piezoelectric wafer actuators bonded to the elastic medium, including analytical, numerical, and hybrid approaches to model the coupled piezo-elastodynamic behavior. Some resulting ultrasonic wave phenomenon and applications relevant to SHM are also summarized. It is demonstrated that the integral model approach [50,62–66] is a good approach to consider the coupled piezo-elastodynamic behavior between the piezoelectric actuator and the host medium and simulate the resulting guided wave propagation, especially for high-frequency cases.
